# Poor supply chain management and stock-outs of point-of-care diagnostic tests in Upper East Region’s primary healthcare clinics, Ghana

**DOI:** 10.1371/journal.pone.0211498

**Published:** 2019-02-27

**Authors:** Desmond Kuupiel, Boikhutso Tlou, Vitalis Bawontuo, Paul K. Drain, Tivani P. Mashamba-Thompson

**Affiliations:** 1 Department of Public Health Medicine, School of Nursing and Public Health, University of KwaZulu-Natal, Durban, South Africa; 2 Faculty of Health and Allied Sciences, Catholic University College of Ghana, Fiapre, Sunyani, Ghana; 3 International Clinical Research Centre, Department of Global Health, University of Washington, Seattle, Washington, United States of America; 4 Division of Infectious Diseases, Department of Medicine, University of Washington, Seattle, Washington, United States of America; 5 Department of Epidemiology, University of Washington, Seattle, Washington, United States of America; University of Cape Town Faculty of Health Sciences, SOUTH AFRICA

## Abstract

**Introduction:**

Several supply chain components are important to sustain point-of-care (POC) testing services in rural settings. To evaluate the availability of POC diagnostic tests in rural Ghana’s primary healthcare (PHC) clinics, we conducted an audit of the supply chain management for POC diagnostic services in rural Upper East Region’s (UER) PHC clinics, Ghana to determine the reasons/causes of POC tests deficiencies.

**Material and methods:**

We conducted a review of accessible POC diagnostics in 100 PHC clinics in UER, Ghana from February to March 2018. We used a monitoring audit tool adopted from the World Health Organization and Management Science for Health guidelines for supply chain management of diagnostics for compliance. We determined a clinic’s compliance with the stipulated guidelines, and a composite compliant score was defined as a percentage rating of 90 to 100%. We used univariate logistic regression analysis in Stata 14 to determine the level of association between supply chain management and the audit variables.

**Results:**

Overall, the composite compliant score of supply chain management for existing POC tests was at 81% (95%CI: 79%–82%). The mean compliance with distribution guidelines was at 93.8% (95%CI: 91.9%–95.6%) the highest score, whilst inventory management scored the lowest, at 53.5% (95%CI: 49.5%–57.5%) compliance. Of the 13 districts in the region, the results showed complete stock-out of blood glucose test in all selected PHC clinics in seven (53.8%) districts, haemoglobin and hepatitis B virus test in three (23.1%), and urine protein test in two (15.4%) districts. Based on our univariate logistics regression models, stock-out of tests at the Regional Medical and District Health Directorates stores in the region, high clinic attendance, lack of documentation of expiry date/expired tests, poor documentation of inventory level, poor monitoring of monthly consumption level, and failure to document unexplained losses of the various POC tests were significant predictors of complete test stock-out in most of the clinics in the Upper East Region.

**Discussion:**

There is poor supply chain management of POC diagnostic tests in UER’s PHC clinics. Improvement in inventory management and human resource capacity for POC testing is critical to ensure accessibility and sustainability of POC diagnostic services in resource-limited settings PHC clinics.

## Introduction

Diagnostics are an essential component to advance universal health coverage, address health emergencies, and promote healthier populations [1]. However, several primary healthcare (PHC) facilities lack sophisticated laboratory infrastructure and do not have the resources to transport clinical specimens to central laboratories, where available, and point-of-care (POC) diagnostics can provide a solution to this challenge [2–4]. The World Health Organization (WHO) has pre-qualified some POC diagnostic technologies for use in resource-limited settings to facilitate POC testing, disease management and prevention [1]. However, supply chain management challenges may hamper the accessibility of these essential POC diagnostics and possibly result in stock-outs, especially in low- and middle-income countries (LMIC) rural clinics such as Ghana [5, 6]. For instance, nearly 50% of clients did not have access to HIV and syphilis testing in Guatemala partially due to test stock-outs [7]; almost half of the rapid syphilis test (RST) implementation pilot sites and a third of rollout sites in Zambia reported test stock-outs [8]. In addition, stock-outs of RSTs were reported at various stages in Zambia in a study aimed at assessing the impact of RSTs and treatment in pregnant women [9]. It is also evident that stock-outs of various POC tests have been reported in primary healthcare facilities in Mozambique [10], Uganda [11], and South Africa [12]. In Ghana, a study aimed at assessing the accessibility of POC diagnostic services for antenatal care in rural primary PHC clinics, revealed poor availability of POC tests [13], which is similar to what has been reported in rural South Africa [14].

Adequate supply chain management prevents diagnostic test stock-outs and sustains POC diagnostic services in rural health facilities [5, 15]. Supply chain management has been defined by various studies to include all activities leading to the production, selection, quantification, negotiation, procurement, quality assurance, storage, inventory management, distribution and redistribution of a service or product [16, 17]. In this study, we define supply chain management as events leading to the selection, distribution, storage and inventory management, as well as human resource capacity, for POC testing in rural primary healthcare clinics. Strict adherence to WHO quality-ASSURED (Affordability, Sensitivity, Specificity, User-friendly, Rapid and robust, Equipment-free and Delivered) criteria for selection of POC diagnostics for rural PHC clinics ensures accessibility of POC tests for rural populations and improves access to healthcare [18]. Timely distribution of POC diagnostic tests, availability of adequate storage facilities, and ensured availability of the needed human resource capacity for POC diagnostics management and testing is important to sustain POC testing [5, 17, 19]. We, therefore, sought to audit the supply chain management for POC diagnostic tests in rural Upper East Region’s (UER) PHC clinics, Ghana to determine the reasons for POC tests deficiencies.

## Material and methods

### Ethical consideration

This study was approved by the Navrongo Health Research Centre Institutional Review Board/Ghana Health Service (approval number: NHRCIRB291) and the University of KwaZulu-Natal Biomedical Research Ethics Committee (approval number: BE565/17). Permission was obtained from the Upper East Regional Health Directorate prior to the conduct of this study. All study participants also signed an informed consent prior to participating in the study.

### Study design

This is a cross-sectional study, which involved an audit of the role of supply chain management of POC diagnostic test accessibility in rural PHC clinics in the UER of Ghana. This current study is a follow-up on a prior study involving 100 rural PHC clinics from all 13 districts aimed at assessing the accessibility of POC diagnostic services for maternal health in rural PHC clinics in the UER [13]. The findings of the previous study demonstrated low availability of POC test (less than 5 tests for most of the clinics. Supply chain management was found to be a major barrier hence; informing our decision to conduct this follow-up study to audit the supply chain management for POC diagnostic tests to determine the reasons for POC tests deficiencies.

### Study area and population

**Fig 1:**
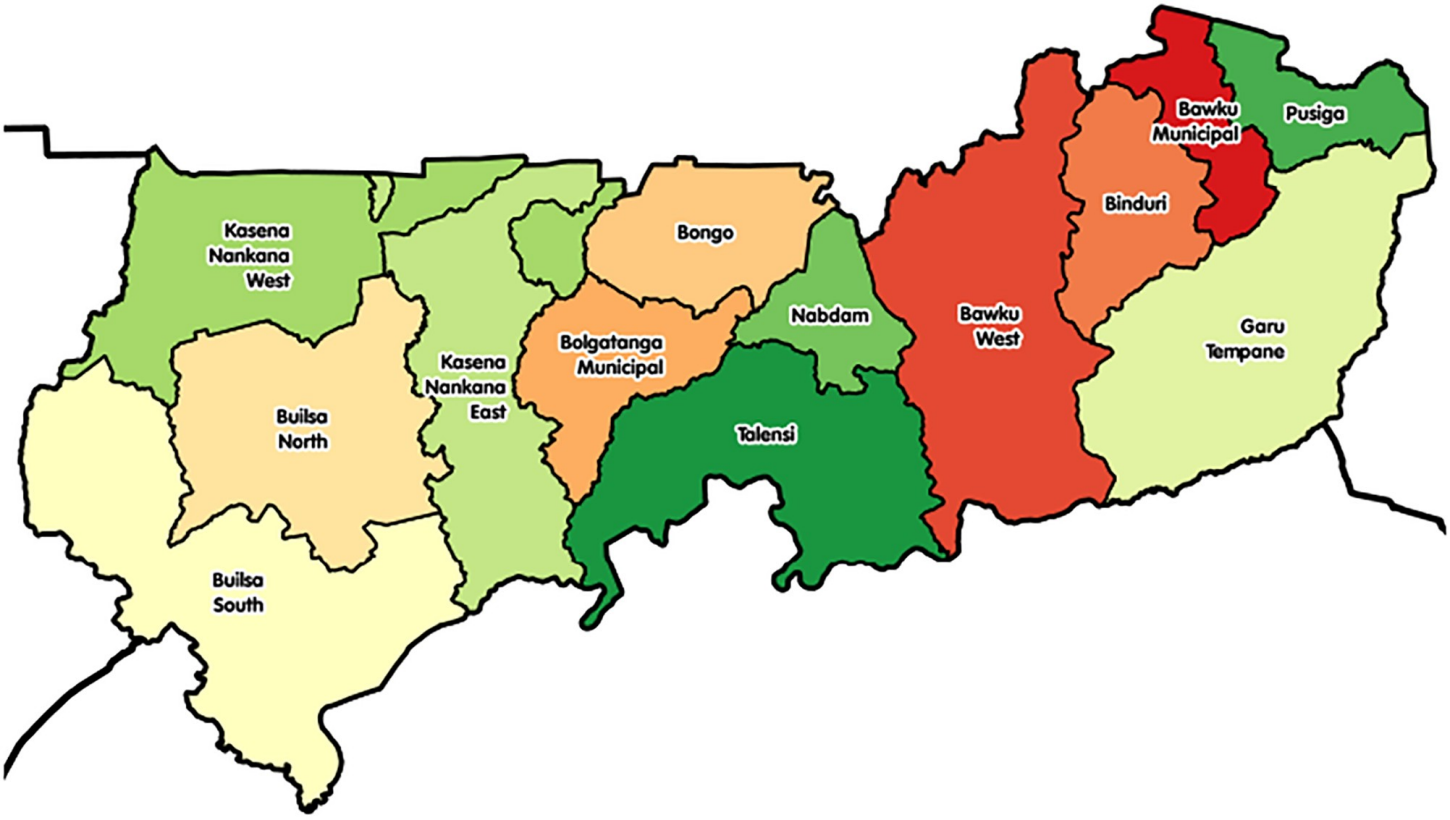
Map of Upper East Region showing the 13 administrative districts.

This study was conducted in the UER of Ghana. The region was chosen because it is the least (21%) urbanized in Ghana with a maternal mortality ratio of 108/100000 live births [20]. It is located in the north-eastern corner of Ghana, bordered by Burkina Faso to the north, Togo and the Upper West Region to the east and west respectively, and the Northern Region to the south. The region had 1188800 people in 2016 and is considered largely (79%) rural and scattered in dispersed settlements [20]. The main source of income for the majority of the population is farming. The region is divided into 13 administrative districts (Fig 1) and all were used in this study.

### Sampling

This was a follow-up study to a prior study aimed at assessing the accessibility of POC diagnostic services for maternal healthcare in rural health facilities in the UER, Ghana [13]. The previous study used a multistage sampling strategy comprising stratified sampling, probability proportionate to size and simple random sampling to select 100 rural PHC clinics with representation from all the thirteen districts in the UER. All 100 PHC clinics were surveyed in this current study.

### Data collection procedure

This study was conducted from February to April 2018, using an audit tool adopted from Management Science for Health (MSH) laboratory diagnostic supply chain management [17] and WHO guidelines for selection of POC tests for rural PHC clinics [18]. The audit tool (S1 Table) was pre-tested in ten non-participating rural PHC clinics in the UER and adjusted to suit the local context based on feedback from respondents. The audit tool consisted of a set of questions, each aimed to assess the selection, inventory management, distribution, and human resource capacity for POC testing in the selected PHC clinics. In order to ensure the accuracy of the audit, the PHC clinics managers/supervisors were informed about the purpose of the audit and the procedures that would be followed. Data on the selection of POC diagnostics was obtained based on the WHO quality-ASSURED criteria for POC diagnostic selection for rural PHC clinics [18]. Data on the distribution of POC diagnostics, POC diagnostics storage and inventory management, and human resource capacity for POC testing were obtained using the audit tool in order to investigate the supply chain management of POC diagnostic tests of the selected rural PHC clinics. We also obtained data on stock levels of eight (8) POC tests: HIV, malaria, syphilis, haemoglobin, urine protein/albumin, urine pregnancy, blood glucose, and hepatitis B, in order to investigate deficiency and determine its relationship with the supply chain management. These tests were the top eight POC tests in the majority of the PHC clinics. These POC tests are also included in the first WHO essential list of diagnostics for resource-limited settings [1]. Finally, we took data on the average clinic attendance per month to facilitate the determination of the relationship between clinic attendance and POC test stock-out.

### Statistical analysis

The primary outcome of this study included supply chain management and stock levels of POC tests in all selected PHC clinics in the UER of Ghana. For supply chain management, one point (100%) was allotted to each question if all the requirements for the question were fulfilled for each of the components. A sum of the scores for each component was obtained to provide the overall percentage score. A score of 90 to 100% was considered “compliant” supply chain management, indicating strong, reliable, and satisfactory compliance with the stipulated guidelines. A rating of less than 90% was considered “non-compliant” supply chain management, indicating unsatisfactory compliance of the clinic to the stipulated MSH and the WHO guidelines. POC test stock-out was measured as either “Yes” or “No” of HIV test, malaria test, syphilis test, haemoglobin test, urine protein/albumin test, urine pregnancy test, blood glucose test, and hepatitis B test. That is, Yes = availability of test, and No = complete stock-out of test. Clinic attendance was measured as: 0–100 patients/clients per month = low attendance and > 100/month = high attendance. Data were analysed using Stata version 14. Frequencies, means, standard deviation as well as 95% confidence intervals (CI) were calculated for all eight POC tests audited. We used Univariate logistic regression to associate the independent (reasons for test stock-outs) variables with the dependent variable (POC tests for HIV, malaria, syphilis, haemoglobin, urine protein/albumin, urine pregnancy, blood glucose, and hepatitis B test) and p<0.05 was reported.

### Ethics statement

Data from this study are the property of the University of KwaZulu-Natal and can be made publicly available. All interested researchers/readers/persons who meet the criteria for access to confidential data can access the dataset via Dr Tivani Mashamba-Thompson, the project supervisor and the Academic Leader (Research) for the School of Nursing and Public Health via this email address: Mashamba-Thompson@ukzn.ac.za. Data access may also be requested from the University of KwaZulu-Natal Biomedical Research Ethics Committee (BREC) from the following contacts: The Chairperson BIOMEDICAL RESEARCH ETHICS ADMINISTRATION Research Office, Westville Campus, Govan Mbeki Building University of KwaZulu-Natal P/Bag X54001, Durban, 4000 KwaZulu-Natal, South Africa Tel.: +27 31 260 4769 Fax: +27 31 260 4609 Email: BREC@ukzn.ac.za.

## Results and discussion

### Clinic characteristics

In 100 PHC clinics, 959 health professionals were found, with Community Health Nurses being the majority (30%) and Dispensary Technicians/Assistants being the minority (2.4%). Antenatal client census per month for a majority (75%) of the clinics was less than 100 (Range: 3–360). Of the eight POC tests audited in the region, the haemoglobin test was available in 26 clinics; blood glucose test in seven clinics; HIV test in 83 clinics; syphilis tests in 23 clinics; hepatitis B in 21 clinics; malaria test in 97 clinics, urine pregnancy test in 91 clinics, and urine protein/albumin test in 20 clinics.

### Supply chain management of existing POC tests

Table 1 shows the supply chain management components and compliance with stipulated guideline by the PHC clinics. Generally, the results of this study demonstrated poor supply chain management of existing POC tests in the UER. The mean score for supply chain management was 81.2% (95%CI: 79.5%–82.9%), ranging from 53.5%–93.8%. Inventory management was shown to be weakest with a mean score, estimated at 53.5% (95%CI: 49.5%–57.5%).

**Table 1:** Compliance of PHC clinics to supply chain management guidelines in the Upper East Region.

Supply chain management (N = 100)	
Inventory management	Percentage score (%)
Presence of updated list of POC tests	82
Availability of storage space	64
Availability of stock/bin cards	85
Documented monthly consumption level	33
Documented inventory levels	44
Documented unexplained losses	26
Documented expiry dates of existing POC tests	16
Availability of inventory control forms/book	81
Set minimum and maximum stock levels to match peak consumption levels.	4
Presence of expired POC diagnostics	37
Presence of compiled list of expired POC diagnostic tests	16
**Selection of POC diagnostics**	
Affordable (Patients are not charged for the use of a POC test device/POC testing is free)	100
Perceived sensitivity (POC diagnostics sensitive with very few false-negatives test results)	84
Perceived specificity (POC diagnostics specific with very few false-positives test results)	72
User-friendly	94
Robust	100
Equipment-free	77
Delivered (Enabled diagnosis and treatment of patients at the clinic)	100
**Distribution of existing POC diagnostic tests**	
Timely supply of diagnostics	90
Cross-checked requisition against supply	100
Checked for differences	100
Asked the supply person to note differences	100
Documented differences	100
Asked the supply person to sign against the requisition supplied	100
Documented supplied POC diagnostics information in ledger book	75
Kept requisition forms/books in a safe place	96
**Human resource capacity for performing POC testing**	
Availability of trained personal for POC testing	98
Availability of standard operating procedures (SOPs) for performing POC tests	81
Availability of SOPs for POC test reagent stock management	76
Availability of protocols for disposal of used POC tests and reagents	76

### Inventory management of existing POC tests

Inventory management at the PHC clinic in Ghana is the ultimate responsibility of the clinic supervisor/manager unless otherwise, delegated to another staff within the clinic. All audited PHC clinics had personnel whose duties included managing POC tests. An updated list of POC tests was available in 82% of the clinics; availability of storage space was 64%, and availability of stock/bin cards was 85%. Additionally, monthly consumption level was documented in 33% of the PHC clinics; with 44% documented inventory levels, and 26% documented unexplained losses. Whilst few (16%) PHCs documented expiry dates of existing POC test kits, a majority (81%) of the PHC clinics had inventory control forms/books. Likewise, only 4% of the PHCs had set minimum and maximum stock levels to match with peak consumption levels. Expired POC diagnostics (haemoglobin, syphilis, and HIV) were found in 37% of the PHC clinics, of which only 16% had a compiled list of the expired POC diagnostic tests.

### Selection of existing POC diagnostic tests

Although PHC clinic staff were not involved in the selection of POC tests but rather higher authorities at the District, Regional, and National levels, our finding showed selected POC tests for the clinics largely were in compliance with WHO quality ASSURED guideline. Our findings showed existing POC diagnostics were affordable, enabled rapid testing and treatment at the first visit, as well as requiring no refrigerated storage (robust) in all 100 PHC clinics. 100% of the existing POC diagnostics were found to be suitable for screening pregnant women and all other patients. On perceived sensitivity and specificity of the POC diagnostic tests, 84% of the clinics said the existing POC diagnostics provided sensitive test results with very few false-negatives, whilst 72% said existing POC diagnostics provided specific test results with very few false-positive. Ninety-four percent (94%) of the existing POC diagnostics were found to be user-friendly, and 77% were simple to perform and required minimal training (equipment-free). The mean score for compliance with WHO guidelines for selection of POC diagnostic tests for resource-limited settings such as PHC clinics was 91% (95%CI: 89%–93%).

### Distribution of existing POC diagnostic tests

Distribution of POC tests to PHC clinics is the responsibility of the health authorities at the Regional medical store and District Health Directorate upon request by the PHC clinics. Timely distribution of POC diagnostic tests to PHC clinics, however, depended on the availability of requested tests at the District Health Directorate or at the regional medical stores in the UER. Of the 100 PHC clinics, 90% received POC diagnostic supplies within 24-hours of requisition whenever the test was available either at the District Health Directorate or at the regional medical stores. All 100 PHC clinics cross-checked requisitions against supply to ensure that POC diagnostics supplied corresponded with the test kits requested, as well as checked for differences. Ninety-seven percent (97%) of the PHCs asked the supply person to note differences. A review of requisition books revealed that all differences were documented in all 100 PHC clinics. Ninety-nine percent (99%) of the PHC clinics asked the supply person to sign against the requisition supplied. Seventy-five percent (75%) of the PHC clinics documented supplied POC diagnostics information into the ledger books and 96% kept requisition forms/books in a safe place. The average score for compliance with distribution guidelines by the PHC clinics was 93.8% (95%CI: 91.9–95.6%).

### Human resource capacity for performing POC testing

Training of PHC clinic staffs to enable them to perform testing onsite is the ultimate duty of the health authorities at the District and Regional level. This study results showed majority (98%) of the PHC health professionals were trained in how to use existing POC diagnostics. Standard operating procedures (SOPs) for performing POC tests were available in 81% of the clinics. However, 24% of the clinics did have SOPs for safe disposal of used POC tests, and neither did they have SOPs for reagent stock level management. The average score for compliance with guidelines by the PHC clinics was 85.7% (95%CI: 81.8%–89.6%).

### Stock levels of POC diagnostic tests

Table 2 shows the distribution of POC test stock levels in each district from the 100 PHC clinics participated in this study. The results showed complete stock-outs of haemoglobin, blood glucose, syphilis, hepatitis B, urine protein POC tests in all selected PHC clinic for some districts in the UER. This study results showed complete stock-out of blood glucose test in seven (53.8%) out of the 13 districts in the region. There were also complete stock-outs of haemoglobin and hepatitis B virus tests in three (23.1%) districts. The study results additionally, showed complete stock-out of urine protein test in all selected PHC clinics two (15.4%) districts.

**Table 2:** Distribution of POC test stock levels in all 100 PHC clinics by district in the Upper East Region.

District	Haemoglobin Number (%)	Blood glucose Number (%)	HIV Number (%)	Syphilis Number (%)	Hepatitis B Number (%)	Malaria Number (%)	Urine pregnancy Number (%)	Urine protein Number (%)
Bongo	84(11.5)	50 (18.7)	1503 (17.1)	202(11)	438(35.2)	1899(9.7)	822(22.6)	540(48.5)
Bawku West	76(10.4)	15(5.6)	620 (7)	66(3.6)	0	1266(6.5)	321(8.8)	108(9.7)
Kasena Nanakana Municipal	90(12.3)	0	1038 (11.8)	312(17)	80(6.4)	1736(8.9)	200(5.5)	88(7.9)
Kasena Nanakana West	56 (7.7)	73 (27.3)	613 (6.9)	360(19.7)	26(2.1)	1240(6.3)	75(2.1)	4(0.4)
Builsa North	0	25(9.4)	261(2.9)	23(1.3)	18(1.5)	1322(6.8)	97(2.7)	2(0.2)
Builsa South	0	0	153(1.7)	15(0.8)	0	1005(5.1)	145(3.9)	28(2.5)
Garu-Tempane	67(9.2)	54 (20.2)	1301(14.7)	129(7)	127(10.2)	2252(11.5)	703(19.4)	153(13.7)
Pusiga	2 (0.3)	50 (18.8)	357(4.1)	34	154(12.4)	2547(13)	380(10.5)	2(0.2)
Bawku Municipal	0	0	799(9.7)	0	0	1502(7.7)	220(6.1)	0
Binduri	107(14.6)	0	430(4.9)	218(11.9)	30(2.4)	325(1.7)	60(1.7)	0
Nabdam	123 (16.8)	0	428(4.9)	250(13.6)	125(10.1)	725(3.7)	215(5.9)	126(11.3)
Talensi	63 (8.6)	0	564(6.4)	31(1.7)	195(15.7)	1375(7)	96(2.6)	13(1.2)
Bolgatanga Municipal	63 (8.6)	0	746(8.5)	192(10.5)	50(4)	2367(12.1)	298(8.2)	50(4.4)
**Total**	731	267	8813	1832	1243	19561	3632	1114

**Fig 2:**
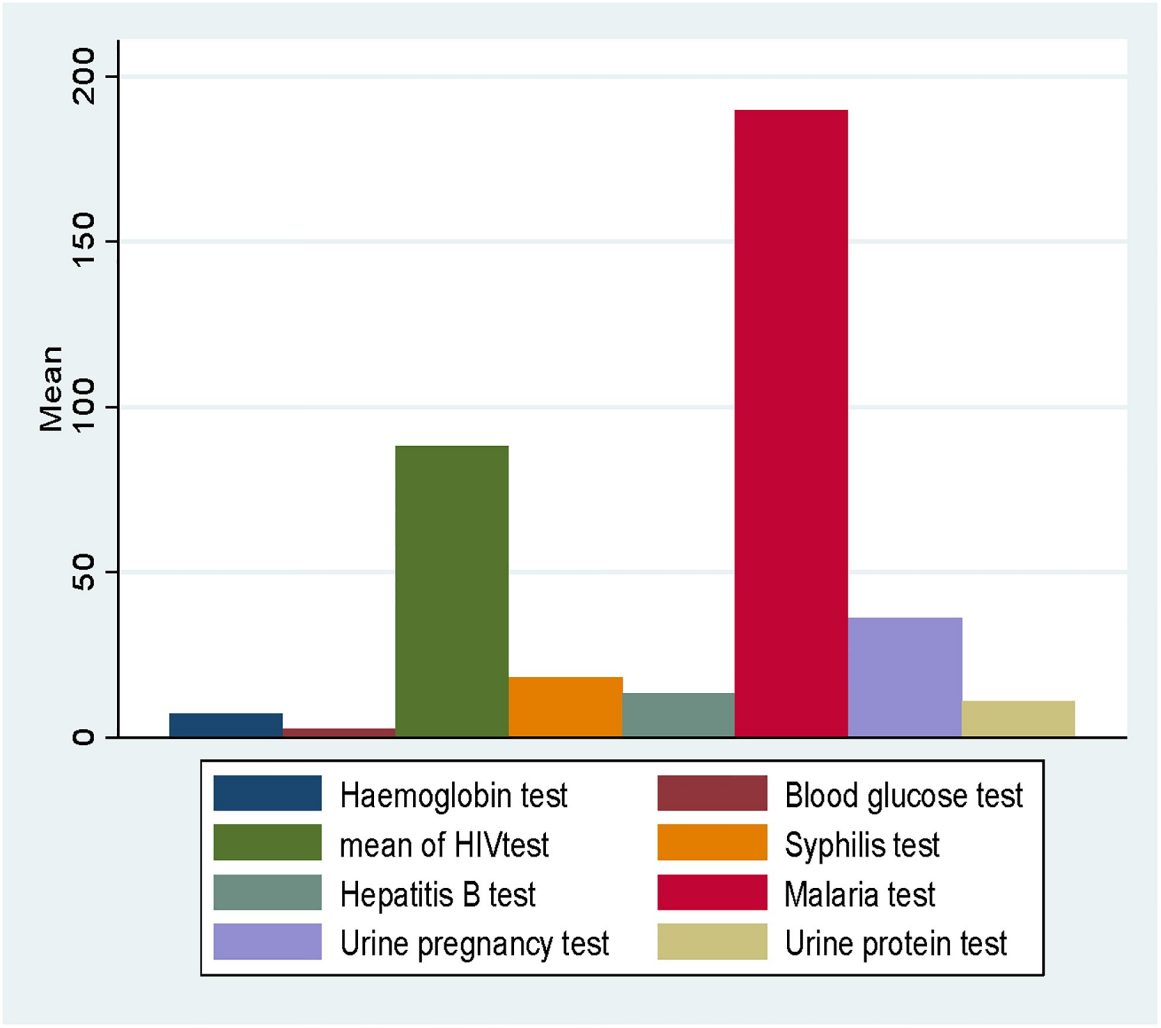
Shows a bar chart of the mean variations in stock levels of POC tests audited in 100 PHC clinics in the Upper East Region.

The results of the audited stock levels of HIV, malaria, syphilis, haemoglobin, urine protein/albumin, urine pregnancy, blood glucose, and hepatitis B POC tests, showed that the mean (plus or minus) standard deviation (SD) stock level of malaria tests in all 100 PHC clinics was 189. 8 tests (SD = 188.8). Blood glucose test was revealed to be the least available in all 100 PHC clinics, with a mean stock level of 2.8 tests (SD = 10.6), as illustrated in Fig 2.

### Causes/Reasons for complete stock-outs of POC diagnostic tests in PHC clinics

Table 3 shows a summary the strength of association between predictors and POC test stock stock-outs. This study results showed that complete stock-outs of POC diagnostic tests in PHC clinics in the UER were largely due to inadequate inventory management and test stock-out at the Regional Medical Store/District Health Directorates. The univariate logistic regression models showed generally, a significant association between inadequate inventory management and stock-out of tests in the PHC clinics (p < 0.001, 95% CI: 1.1–1.2). Complete stock-out of haemoglobin, blood glucose, syphilis, hepatitis B, urine pregnancy, and urine protein tests additionally showed significant associated with lack of tests at the various storage points in the UER with all p<0.05. The results also demonstrated that reasons such as lack of microcuvette for the HaemoCue device, inadequate documentation of consumption level, and failure to document expiry dates of POC diagnostics separately showed significant association with complete POC test stock-outs in the PHC clinics in the UER. Haemoglobin, blood glucose, HIV, syphilis, and hepatitis B POC tests stock levels were significantly associated with clinic attendance.

**Table 3:** Summarised results of the strength of association between predictors and POC test stock-outs in the Upper East Region’s rural PHC clinics.

**Haemoglobin test**	**Odds Ratio**	**95% Confidence Interval**	**p-value**
Lack of micro cuvette in Regional/District stores	0.35	0.22–0.55	< 0.001
No documentation of expiry date	0.14	0.04–0.45	0.001
No documentation of inventory level	0.03	0.01–0.14	< 0.001
No documentation of unexplained losses	0.27	0.10–0.72	0.008
No documentation of monthly consumption level	0.15	0.05–0.39	< 0.001
High clinic attendance	1.00	1.00–1.01	0.023
**Blood glucose test**	**Odds Ratio**	**95% Confidence Interval**	**p-value**
Lack of test in Regional/District stores	0.08	0.03–0.16	< 0.001
No documentation of minimum and maximum stock level	0.08	0.04–0.17	< 0.001
High clinic attendance	1.02	1.00–1.03	0.001
**HIV**	**Odds Ratio**	**95% Confidence Interval**	**p-value**
No documentation of expiry date	3.94	2.31–6.71	< 0.001
No documentation of inventory level	0.06	0.01–0.46	0.007
No documentation of unexplained losses	0.35	1.95–5.76	< 0.001
No documentation minimum and maximum stock level	4.65	2.75–7.85	< 0.001
High clinic attendance	1.03	1.00–1.05	0.012
**Syphilis**	**Odds Ratio**	**95% Confidence Interval**	**p-value**
Lack of test in Regional/District stores	0.49	0.32–0.75	0.001
No documentation of inventory level	0.30	0.12–0.71	0.007
No documentation of monthly consumption level	0.24	0.01–0.59	0.002
High clinic attendance	1.01	1.01–1.02	0.001
**Hepatitis B test**	**Odds Ratio**	**95% Confidence Interval**	**p-value**
Lack of test in Regional/District stores	0.27	0.16–0.43	< 0.001
No documentation of inventory level	0.31	0.11–0.84	0.022
No documentation of monthly consumption level	0.35	0.13–0.94	0.038
High clinic attendance	1.01	1.00–1.02	0.004
**Malaria test**	**Odds Ratio**	**95% Confidence Interval**	**p-value**
No documentation of expiry date	27	8.53–85.47	< 0.001
No documentation of inventory level	17.67	5.52–56.53	< 0.001
No documentation of unexplained losses	23.67	7.45–73.14	< 0.001
No documentation of minimum and maximum stock level	31	9.82–97.87	< 0.001
**Urine pregnancy test**	**Odds Ratio**	**95% Confidence Interval**	**p-value**
Lack of test in Regional/District stores	10.11	5.09–20.06	< 0.001
No documentation of expiry date	8.33	4.17–16.64	< 0.001
No documentation of unexplained losses	7.22	3.59–14.50	< 0.001
No documentation of minimum and maximum stock level	9.67	4.87–19.20	< 0.001
**Urine protein test**	**Odds Ratio**	**95% Confidence Interval**	**p-value**
Lack of test in Regional/District stores	0.23	0.14–0.39	< 0.001
No documentation of inventory level	0.15	0.46–0.50	0.002

Our multivariate regression analysis model (S2 Table) also showed haemoglobin test stock level was significantly associated with documentation of expiry date, documentation of inventory level, documentation of unexplained losses, documentation of monthly consumption level, and documentation of minimum and maximum stock level (all p<0.05). Stock level of HIV, malaria, and blood glucose tests were shown to be significantly associated with only clinic attendance (all p<0.001), whilst syphilis test stock level was only significantly associated with documentation of expiry date, documentation of monthly consumption level, and documentation of minimum and maximum stock level (all p<0.001). Hepatitis B stock level was also found to be significantly associated with only documentation of unexplained losses (p = 0.011) and documentation of minimum and maximum stock level (p<0.001). The results of the multivariate regression analysis further showed urine pregnancy test stock level was only significantly associated documentation of minimum and maximum stock level (p<0.001), and urine protein test dipsticks stock level was only significantly associated with documentation of inventory level (p = 0.015), and documentation of minimum and maximum stock level (p<0.001).

## Discussion

We audited the supply chain management of POC diagnostics in rural PHC clinics in the UER of Ghana. In this study, inadequate supply chain management of POC diagnostics was high, and the combined compliance score was strong on distribution and selection of POC diagnostics. POC diagnostics inventory management was revealed to be weak, followed by human resource capacity for POC testing in the region. This audit also demonstrated stock-outs of haemoglobin, blood glucose, syphilis, hepatitis B, and urine protein/albumin POC test kits in the majority of the rural clinics. Lack of POC tests at the Regional/District Health Directorate stores, inadequate inventory management, and high clinic attendance demonstrated significant association with test stock-out in the PHC clinics.

Supply chain management challenges for POC diagnostics have been shown in other studies within LMICs [5, 15, 21–23]. Hawkes et al. (2011), Jani et al. (2013), Tirinato et al. (2007) and Palamontain et al. (2012), in their various studies, identified weaknesses in POC diagnostics supply chain management that included human resources and training for POC testing [24–27]. Weak supply chain management is very challenging to achieving cost-efficient implementation of POC diagnostics [27, 28]. It has been one of the major causes of poor stock levels of POC tests in LMICs [29]. Additionally, poor supply chain management leads to downtime of POC diagnostic device operating times [30]. It can potentially result either in stock-outs of POC tests or in over-stocking of tests beyond PHC clinic consumption level which may expire [30]. Ansbro et al. (2015) and Bonawitz et al.’s (2015) studies in Zambia demonstrated a high level of syphilis POC test stock-outs at rollout sites, and at various stages of the implementation for pregnant women, as well as at the evaluation stage [8, 9]. Smith et al.’s (2015) study conducted in Guatemala’s rural antenatal clinics also showed evidence of stock-outs of HIV, syphilis, and hepatitis B virus POC tests [7]. Dassah et al.’s (2018) study conducted in Ghana aimed at exploring healthcare providers’ experiences and challenges in antenatal syphilis screening following the national rollout of rapid syphilis POC tests further reported frequent stock-outs of syphilis and HIV tests in the health facilities [31]. Another example is Jaya et al.’s (2017) audit in rural KwaZulu-Natal PHC clinics, which indicated that four out of 11 rural clinics reported past experiences of HIV rapid test kit stock-outs [12]. Contrary to our findings, a study by Hasselback et al. (2014) in Capo Delgado province in Mozambique revealed substantially high levels of malaria POC test stock-outs in rural health facilities with increasing levels of consumption in the region [10].

Studies have demonstrated reasons of POC test stock-outs to include inadequate/underestimation of supply chain management during implementation [6, 8, 7, 29]; inaccurate documentations and distribution systems [10, 23]; lack of storage space [11]; and poor commodity management [9] which support these study findings. To address stock-outs of test POC tests Peeling and Ronald (2009) recommended among other good supply-chain management, effective training, and information systems [29]. Electronic connectivity such as the use of dashboards to prevent stock-outs and also detect high consumption and low consumption clinics to support redistribution of tests to prevent the expiration of tests has been suggested [23]. Tirinato et al. (2007) suggested the use of point-of-care inventory management system [24]. Practical training of health workers on how to perform POC tests and interpret of the results, stock management, record keeping, and quality control have also been found to improve test availability for use by rural health workers [21]. It is therefore imperative for Ghana to adopt an electronic inventory management system, for PHC clinics alongside existing strategies and ongoing activities such as the National Health Insurance Scheme, provision of equipment to existing health facilities at all levels, building of Community-Based Planning and Services (CHPS) compounds, and training and posting more health professions to help achieve Universal Health Coverage.

The use of the WHO quality ASSURED criteria [18] and the MSH guidelines for diagnostic supply chain management [17] has enabled us to adequately audit the supply chain management for POC diagnostic services in rural PHC clinics in Ghana. Auditing the supply chain management systems, especially in these rural health facilities, provides an opportunity to streamline measures to ensure equity in the demand and supply of POC diagnostics [15]. The study findings have contributed to gaining a better understanding of the POC diagnostic supply chain management in these clinics and revealed areas that require attention to improve the accessibility of POC diagnostic services to patients. The findings have also provided evidence-based information to help with planning and improving the access to these services in rural health facilities. They are additionally useful in helping to plan and achieve universal coverage as well as the health-related sustainable development goals. These findings may be generalizable to other resource-limited settings.

There were several study limitations. We noticed that the supply of POC diagnostics to PHC health facilities in Ghana depended on the availability of the tests at the national and regional medical stores as well as at the district health directorate stores, which was not considered in our study. We recommend a study to audit the availability, stock level, reasons for stock-outs of tests at the District and Regional levels to ensure sustainability of POC testing services in PHC clinics. We also realized that selection and procurement of POC diagnostics is done at the national level and could be constrained by lack of funding and which may also affect the availability of POC tests in rural health facilities. On the performance of the available POC tests, we were limited to measure only the perceived sensitivity and specificity of the tests since PHC health workers were more unlikely to have access to confirmatory testing results to know the diagnostic accuracy of POC tests. Global development, procurement, and forecasting may also play a larger role for POC diagnostics supply chains because the production lines for new assays entering the market are mostly unable to meet the demand of rapid recommendations that lead to rapid global uptake [27, 28]. In view of this, we also recommend increased development of POC diagnostics in LMICs in order to meet demand and supply. This study did not investigate the root causes responsible for the poor inventory management although the majority of the study respondents said they were trained on inventory management. We recommend a study to unravel the root causes/reasons accounting for the poor inventory management at the PHC clinics in the region in order to sustain POC testing services.

Accessibility to POC testing in rural PHC clinics is mostly limited and supply chain management systems are mostly poor and unable to meet demand [32]. Improving supply chain management, especially inventory management, and training of healthcare workers would sustain accessibility of POC diagnostic services and may ultimately lead to sustained health system strengthening [21]. We recommend regularly refresher training/workshops for PHC clinic staffs responsible for POC diagnostic inventory management as well as their subordinates particularly, new staffs. Documentation of minimum and maximum monthly consumption levels, inventory levels, unexplained losses, expiry dates of existing POC tests ensure sufficient availability of POC tests in rural health facilities [24]. We also recommend an electronic system of inventory management to help detect PHC clinics with high and low consumption levels and to redistribute POC tests to high consumption clinics in order to prevent test kits from expiring [6, 33].

Although this study finding suggested the distribution of existing POC tests to be adequately managed, documentation challenges by PHC staffs were revealed. We recommend that the District/Regional health authorities should regularly organize refresher training for PHC staff on documentation of test stock levels on site to aid forecasting demand to ensure continued supply of the diagnostic tests to match consumption. We additionally recommend the adoption of the proposed lean and agile supply chain management framework for POC diagnostics in LMICs [5] to suit local contexts in order to ensure universal health coverage and improve health outcomes in rural communities.

## Conclusion

There is poor supply chain management of POC diagnostics in the Upper East Region’s rural PHC clinics. The audit results have shown higher deficiencies in inventory management and human resource capacity for POC diagnostic services in audited PHC clinics in rural UER. Improving inventory management, training of healthcare workers, and provision of standard operating procedures, alongside increased procurement of POC diagnostics is highly recommended strengthening rural healthcare delivery and outcomes. Finally, supply chain management strategies for POC diagnostics need to be well planned to ensure accessibility of POC diagnostic services in rural resource-limited settings, which could ultimately lead to universal health coverage.

## Supporting information

S1 Table Audit tool. (DOCX)

S2 Table Multivariate regression analysis output from Stata 14. (DOCX)

S1 Dataset SCM data.xlsx. (XLSX)

## Abbreviations

LMICs: Low- and middle income countries
MSH: Management Science for Health
PHC: Primary Healthcare
POC: Point-of-Care
UER: Upper East Region
WHO: World Health Organization
