# Supplementary material for: Poor supply chain management and stock-outs of point-of-care diagnostic tests in Upper East Region’s primary healthcare clinics, Ghana
Source: PLoS One. 2019 Feb 27;14(2):e0211498. doi: 10.1371/journal.pone.0211498 (PMC6392218; doi:10.1371/journal.pone.0211498)
Supplement: S1 Table — (DOCX) [file pone.0211498.s001.docx]

**S1 Table: Supply Chain audit tool**

| ***University of KwaZulu Natal, Durban, School of Nursing and Public Health, Discipline of Public Health Medicine***  ***Title:*** *Assessing the accessibility of Antenatal Clinic point of care (POC) diagnostics services in rural Ghana* |
| --- |

| **Level code:** | **Date:** |
| --- | --- |
| **Name of clinic:** |  |
| **Assessors name:** |  |

| **Inventory management** | **Yes** | **No** | **Comment** |
| --- | --- | --- | --- |
| 1. Availability of personnel whose duties include management of existing POC diagnostics at the facility? |  |  |  |
| 1. Presence of updated list of existing POC diagnostics in the last three months? |  |  |  |
| 1. Document expiring dates of existing POC diagnostics |  |  |  |
| 1. Document inventory levels for POC diagnostics |  |  |  |
| 1. Document unexplained losses (leakage) of POC diagnostics |  |  |  |
| 1. Availability of storage conditions such as light, temperature, and sanitation, for test kits and diagnostic reagents |  |  |  |
| 1. Availability of computerized or manual recorded inventory |  |  |  |
| 1. Availability of basic records cards such as stock or bin cards |  |  |  |
| 1. Availability of monthly consumption records |  |  |  |
| 1. Availability of inventory control forms |  |  |  |
| 1. Availability of expired POC diagnostics |  |  |  |
| 1. Compile list of expired POC diagnostics |  |  |  |
| **Selection** |  |  |  |
| 1. Involve managers/persons responsible for POC diagnostics in the selection of POC diagnostics for the facility? |  |  |  |
| 1. Are existing POC diagnostics affordable? |  |  |  |
| 1. Are these diagnostics sensitive with very few false-negatives? |  |  |  |
| 1. Are these diagnostics specific with very few false-positives? |  |  |  |
| 1. Are existing POC diagnostics user –friendly (simple to perform and requiring minimal training)? |  |  |  |
| 1. Do existing POC diagnostics enable rapid testing and treatment rapid at first visit? |  |  |  |
| 1. Are these diagnostics robust, for example not requiring refrigerated storage |  |  |  |
| 1. Do existing POC diagnostics equipment-free? |  |  |  |
| 1. Are these diagnostics suitable for antenatal screening (delivered to those who need it)? |  |  |  |
| **Stock level** | **Yes** | **No** | **Quantity** |
| 1. Are there POC diagnostics for the following at the facility? |  |  |  |
| 1. HIV |  |  |  |
| 1. Hepatitis B |  |  |  |
| 1. Hepatitis C |  |  |  |
| 1. Syphilis |  |  |  |
| 1. G6PD |  |  |  |
| 1. Malaria |  |  |  |
| 1. Diabetes |  |  |  |
| 1. Haemoglobin |  |  |  |
| 1. Sickling |  |  |  |
| 1. Rhesus |  |  |  |
| 1. Tuberculosis |  |  |  |
| 1. Urine pregnancy test |  |  |  |
| 1. Chlamydia |  |  |  |
| 1. Gonorrhoea |  |  |  |
| 1. Urine albumin/total protein |  |  |  |
| 1. Faecal occult blood/ calprotectin |  |  |  |
| **Others** |  |  |  |
|  |  |  |  |
|  |  |  |  |
|  |  |  |  |
| **Supply and distribution** | **Yes** | **No** | **Comment** |
| 1. How often is existing POC diagnostics requisition made? (Please choose only one option) |  |  |  |
| 1. Daily |  |  |  |
| 1. Weekly |  |  |  |
| 1. Monthly |  |  |  |
| 1. Quarterly |  |  |  |
| 1. Every 6 months |  |  |  |
| 1. Annually |  |  |  |
| 1. How often are existing POC diagnostics supplied following requisition? (Please choose only one) |  |  |  |
| 1. Daily |  |  |  |
| 1. Weekly |  |  |  |
| 1. Monthly |  |  |  |
| 1. Quarterly |  |  |  |
| 1. Every 6 months |  |  |  |
| 1. Annually |  |  |  |
| 1. Check the delivery form that came with the supplies? |  |  |  |
| 1. Check the supplies against the delivery form and the requisition book? |  |  |  |
| 1. Ask the driver or delivery person to note any difference? |  |  |  |
| 1. Ask the delivery person to sign the accompany form before leaving your facility? |  |  |  |
| 1. Write down delivery information in a ledger book? |  |  |  |
| 1. Write down delivery information in a ledger book? |  |  |  |
| 1. Keep all delivery forms in a safe place? |  |  |  |
| 1. Document all differences? |  |  |  |
| 1. Does a store room for POC diagnostics exist? |  |  |  |
| 1. Do any of the diagnostics have special storage requirements? |  |  |  |
| 1. If yes, are storage facilities available? |  |  |  |
| 1. Is the freezer, refrigerated, or cold storage space adequate? |  |  |  |
| 1. Is the electricity supply reliable? |  |  |  |
| 1. Is there a procedure in place for a cold chain to maintain and monitor special storage temperatures from delivery to storage and use? |  |  |  |
| 1. Are there cold boxes or icepacks for transporting heat-sensitive POC diagnostics? |  |  |  |
| 1. Is there a system in place to monitor freezer, refrigerator, and storeroom temperatures regularly? |  |  |  |
| **Human Resource Capacity** |  |  |  |
| 1. Are users trained to use existing POC diagnostics appropriately? |  |  |  |
| 1. Do they need training updates for new POC testing or procedures? |  |  |  |
| 1. Are approved, written standard operating procedures (SOP) available for performing each POC test? |  |  |  |
| 1. Availability SOP available for stock (Reagents) level management for existing POC diagnostics? |  |  |  |
| 1. Does an SOP available for safe disposal of existing POC diagnostics? |  |  |  |

**Thank for your participation and cooperation**
