# Supplementary material for: Poor supply chain management and stock-outs of point-of-care diagnostic tests in Upper East Region’s primary healthcare clinics, Ghana
Source: PLoS One. 2019 Feb 27;14(2):e0211498. doi: 10.1371/journal.pone.0211498 (PMC6392218; doi:10.1371/journal.pone.0211498)
Supplement: S2 Table — (DOCX) [file pone.0211498.s002.docx]

**S2 Table: Multivariate regression analysis output from Stata 14**

_ ____ ____ ____ ____ (R)

/__ / ____/ / ____/

___/ / /___/ / /___/ 14.0 Copyright 1985-2015 StataCorp LP

Statistics/Data Analysis StataCorp

4905 Lakeway Drive

Special Edition College Station, Texas 77845 USA

800-STATA-PC http://www.stata.com

979-696-4600 stata@stata.com

979-696-4601 (fax)

Single-user Stata perpetual license:

Serial number: 401406221503

Licensed to: Emmanuel Nakua

PMA2020

Notes:

1. Unicode is supported; see help unicode_advice.

2. Maximum number of variables is set to 5000; see help set_maxvar.

3. New update available; type -update all-

import excel "C:\Users\pc\Desktop\Supplementary data- Survey\SCM data file\SCM data 2 - C.xlsx", sheet("ABC") firstrow

. mvreg Haemoglobintest Bloodglucosetest HIVtest Syphilistest Hepatitisbtest Malariatest Urinepregnancytest Urineproteintest = Clinicattendancem

> onth Documentsexpiringdates Documentinventorylevels Documentunexplainedlosses DocumentationofmonthlyconsumptlevelDocumentationofminimumandmaximumstockle

Equation Obs Parms RMSE "R-sq" F P

Haemoglobi~t 100 7 11.72442 0.4833 14.49784 0.0000

Bloodgluco~t 100 7 9.35588 0.2654 5.600947 0.0001

HIVtest 100 7 97.66803 0.1549 2.840049 0.0139

Syphilistest 100 7 31.48634 0.4812 14.37452 0.0000

Hepatitisb~t 100 7 43.35034 0.2563 5.343087 0.0001

Malariatest 100 7 159.5943 0.3285 7.584067 0.0000

Urinepregn~t 100 7 71.55342 0.2482 5.117224 0.0001

Urineprote~t 100 7 44.71512 0.3173 7.205019 0.0000

Coef. Std. Err. t P>t [95% Conf. Interval]

Haemoglobintest

Clinicattendancemonth .0380988 .0190712 2.00 0.049 .0002272 .0759703

Documentsexpiringdates -19.83186 3.774088 -5.25 0.000 -27.32645 -12.33727

Documentinventorylevels -8.316225 3.344553 -2.49 0.015 -14.95784 -1.674607

Documentunexplainedlosses 8.191117 3.516076 2.33 0.022 1.208887 15.17335

Availabilityofmonthlyconsumpt -7.215281 2.808807 -2.57 0.012 -12.79302 -1.637546

Setminimumandmaximumstockle -16.48333 6.176561 -2.67 0.009 -28.74876 -4.217904

_cons 84.40562 14.41061 5.86 0.000 55.789 113.0222

Bloodglucosetest

ANCattendancemonth .0830601 .0152185 5.46 0.000 .0528392 .1132809

Documentsexpiringdates .6524649 3.011655 0.22 0.829 -5.328085 6.633015

Documentinventorylevels -2.423496 2.668893 -0.91 0.366 -7.723389 2.876398

Documentunexplainedlosses 4.106914 2.805766 1.46 0.147 -1.464781 9.678609

Availabilityofmonthlyconsumpt 2.121164 2.241378 0.95 0.346 -2.329769 6.572097

Setminimumandmaximumstockle 6.534192 4.928786 1.33 0.188 -3.2534 16.32178

_cons -23.63587 11.49941 -2.06 0.043 -46.47142 -.8003229

HIVtest

ANCattendancemonth .5536847 .1588687 3.49 0.001 .2382029 .8691664

Documentsexpiringdates -14.60139 31.43931 -0.46 0.643 -77.03363 47.83085

Documentinventorylevels -25.52491 27.86115 -0.92 0.362 -80.85163 29.80181

Documentunexplainedlosses 51.83511 29.28999 1.77 0.080 -6.329009 109.9992

Availabilityofmonthlyconsumpt -16.24529 23.39823 -0.69 0.489 -62.70954 30.21895

Setminimumandmaximumstockle 13.73239 51.45265 0.27 0.790 -88.44238 115.9072

_cons 28.90234 120.0448 0.24 0.810 -209.4829 267.2875

Syphilistest

ANCattendancemonth .0648622 .0512163 1.27 0.209 -.0368432 .1665676

Documentsexpiringdates -37.64779 10.13544 -3.71 0.000 -57.77477 -17.52081

Documentinventorylevels -4.338782 8.981912 -0.48 0.630 -22.17508 13.49752

Documentunexplainedlosses 6.248521 9.442544 0.66 0.510 -12.5025 24.99954

Availabilityofmonthlyconsumpt -25.20489 7.54315 -3.34 0.001 -40.18409 -10.22569

Setminimumandmaximumstockle -97.66954 16.58737 -5.89 0.000 -130.6088 -64.73031

_cons 312.8029 38.70019 8.08 0.000 235.952 389.6538

Hepatitisbtest

ANCattendancemonth .0757089 .0705145 1.07 0.286 -.064319 .2157367

Documentsexpiringdates -3.092798 13.95446 -0.22 0.825 -30.80359 24.618

Documentinventorylevels -25.12506 12.36628 -2.03 0.045 -49.68205 -.5680767

Documentunexplainedlosses 33.64551 13.00048 2.59 0.011 7.829131 59.46188

Availabilityofmonthlyconsumpt -11.49363 10.3854 -1.11 0.271 -32.11697 9.129708

Setminimumandmaximumstockle -91.99239 22.83746 -4.03 0.000 -137.3431 -46.64172

_cons 194.3586 53.28236 3.65 0.000 88.5504 300.1668

Malariatest

ANCattendancemonth 1.576456 .2595991 6.07 0.000 1.060943 2.091968

Documentsexpiringdates 8.365878 51.37335 0.16 0.871 -93.65142 110.3832

Documentinventorylevels 15.02015 45.52646 0.33 0.742 -75.38638 105.4267

Documentunexplainedlosses 20.58867 47.86126 0.43 0.668 -74.45431 115.6317

Availabilityofmonthlyconsumpt -48.87017 38.23383 -1.28 0.204 -124.795 27.05465

Setminimumandmaximumstockle 74.24724 84.07612 0.88 0.379 -92.71126 241.2057

_cons -51.03231 196.159 -0.26 0.795 -440.5652 338.5006

Urinepregnancytest

ANCattendancemonth .1768284 .1163902 1.52 0.132 -.0542994 .4079562

Documentsexpiringdates 4.887824 23.03302 0.21 0.832 -40.8512 50.62685

Documentinventorylevels -27.4592 20.4116 -1.35 0.182 -67.99259 13.07418

Documentunexplainedlosses 16.65868 21.45839 0.78 0.440 -25.95344 59.2708

Availabilityofmonthlyconsumpt -12.28331 17.14198 -0.72 0.475 -46.32388 21.75727

Setminimumandmaximumstockle -160.1326 37.69517 -4.25 0.000 -234.9877 -85.27747

_cons 364.0735 87.94706 4.14 0.000 189.4281 538.719

Urineproteintest

ANCattendancemonth -.0289414 .0727345 -0.40 0.692 -.1733776 .1154949

Documentsexpiringdates 4.233679 14.39378 0.29 0.769 -24.34952 32.81688

Documentinventorylevels -31.65477 12.7556 -2.48 0.015 -56.98487 -6.324667

Documentunexplainedlosses 26.35311 13.40977 1.97 0.052 -.2760324 52.98225

Availabilityofmonthlyconsumpt -13.04021 10.71235 -1.22 0.227 -34.31282 8.232401

Setminimumandmaximumstockle -125.3201 23.55644 -5.32 0.000 -172.0986 -78.54172

_cons 276.11 54.95982 5.02 0.000 166.9707 385.2493

.
